# Unbalanced Macronutrient Intakes, Multiple Micronutrient Inadequacies, and Diarrhea Underscore Low-Height-for-Age in Indigenous Panamanian Preschool Children

**DOI:** 10.1016/j.cdnut.2025.107547

**Published:** 2025-09-04

**Authors:** Marilyn E Scott, Dorian Irwin Kristmanson, Eduardo Ortega-Barria, Kristine G Koski

**Affiliations:** 1 Institute of Parasitology, McGill University (Macdonald Campus), Sainte-Anne-de-Bellevue, Quebec, Canada; 2 School of Human Nutrition, McGill University (Macdonald Campus), Sainte-Anne-de-Bellevue, Quebec, Canada; 3 Secretaria Nacional de Ciencia, Tecnología e Innovación, Ciudad del Saber, Republicá de Panamá

**Keywords:** HAZ, preschool children, food intakes, estimated nutrient intakes, diarrhea, Ascaris, fats/oils, sugar, stunting

## Abstract

**Background:**

In remote indigenous Panamanian subsistence farming communities, poor diet, diarrhea, and *Ascaris* may have seasonally distinct contributions to high rates of stunting in preschool children.

**Objectives:**

This study explored the relative contributions of food, nutrient intakes, and infections (diarrhea and intestinal nematodes) to height-for-age *z*-scores (HAZ) during a dry and rainy season in Panama’s Comarca Ngäbe-Buglé.

**Methods:**

This prospective community-based study collected sociodemographic and health data from 328, 12‒59-mo-old children in both the dry and rainy seasons. Diets were assessed in nonbreastfeeding children using a food frequency questionnaire and a 24-h recall during both seasons. Bivariate comparisons between stunted and nonstunted children and between the dry and rainy seasons were conducted. Stepwise linear regression models identified associations of sociodemographic status, infections, food groups, and estimated nutrient intakes with HAZ.

**Results:**

The diet characterized as low fat, high sugar was deficient in micronutrients. Food and nutrient intakes were lower in the rainy than the dry season (*P* < 0.05), and stunted children had fewer servings of dairy than nonstunted children (*P* < 0.05). The incidence of diarrhea was higher in the rainy season (*P* < 0.05). Determinants of HAZ differed between dry and rainy seasons. In dry season models (*P* < 0.0001), HAZ was positively associated with income and fat intake and negatively associated with total sugar intake. In contrast, rainy season models (*P* < 0.0001) revealed that servings of grains/cereals and fat were positively associated with HAZ, and fruit and total sugar intake and diarrhea were negatively associated with HAZ.

**Conclusions:**

The multifactorial nature of linear growth faltering in these preschool children differed by season. The negative impact of diarrhea emerged only in the rainy season, but the negative impact of sugar intake and the positive impact of fat intake emerged in both seasons.

## Introduction

Preschool children are particularly vulnerable to stunting [height-for-age *z*-score (HAZ) <‒2 SD], a physiological process of linear growth faltering that often begins in utero [1,2]. The current United Nations’ Sustainable Development Goals [3] target the elimination of stunting and wasting in all children younger than 5 y by 2030 [4], and recognize strong regional disparities [5]. Between 1980 and 2000, the prevalence of stunting in Latin America had decreased by one-half, but only a small fraction of this decline occurred in Central America, where stunting was the dominant anthropometric indicator of malnutrition [6]. Stunting in Central America continues to be prevalent, and Guatemala has the third highest prevalence (∼46%) among 56 low- and middle-income countries (LMIC) [5]. In 2003, the Panamanian Ministry of Health (MoH) reported stunting in 21% of children under 5 but 60% in rural indigenous regions [7].

Indigenous ethnicity [8,9], low family income and maternal education [10], limited access to health care [11], short birth spacing [12], low birth weight [13], parental psychological distress [14], inadequate housing and poor access to clean water [15], and floods [16] which disproportionally affect preschool children, have been identified as important sociodemographic, maternal and environmental risk factors for child stunting. A recent Peruvian study concluded that the 75% reduction in stunting in Peruvian children under 5 between 2000 and 2016 was attributed to improved parental education, maternal nutrition, and maternal and newborn health care [17].

Stunting has also been associated with intestinal nematodes [18] in part because *Ascaris* impairs absorption of vitamin A and digestion of fat and lactose [19,20] and because both mega-dose vitamin A [21] and dietary carotenoid administration through biscuits [22] lower reinfection following anthelmintic treatment. A recent meta-analysis concluded that *Ascaris* infection contributed to low HAZ [18], and a recent systematic review reported that the presence of *Ascaris* increased the odds of stunting (odds ratio 2.90, *P* = 0.03) [23]. It has also been suggested that temperature and rainfall may have a strong impact on *Ascaris* transmission [24]. Transmission occurs from ingestion of eggs that withstand harsh environmental conditions in soil [25] and that stick to the surface of unwashed fruits and leafy green vegetables [26,27]. Importantly, Hall et al. [28] highlighted that children need to have diets sufficient to support growth in order for anthelmintic treatment to improve linear growth.

Among infections, diarrhea caused by bacterial, viral, and protozoan parasites leads to stunting [29] due to damage to the intestinal mucosa and the resulting malabsorption of macronutrients and micronutrients, including vitamin A, as well as carbohydrates, fats, and zinc [30]. The incidence of diarrheal disease varies with season [31] and is often more frequent with heavy rainfall in the rainy season [32]. Diarrheal incidence can also be high during water insecurity in the dry season, when reduced water supply limits sanitation and hygiene practices [33].

Micronutrient deficiencies are common in LMIC countries, largely owing to inadequate consumption of food, unbalanced dietary patterns, a lack of dietary diversity [[34], [35], [36], [37]], and poor micronutrient absorption that accompanies gastrointestinal nematode infections and diarrhea [30,38]. In 2008, Bhutta et al. [39] identified several interventions capable of reducing the prevalence of stunting in countries where over 90% of preschool children were stunted. Successful interventions included improved maternal nutrition and health education, breastfeeding and complementary feeding practices, preventive supplementation of micronutrients (vitamin A, zinc, and iron), deworming for intestinal nematode infections, and control of diarrheal disease [39]. Since then, several additional meta-analyses have shown that linear growth is improved when micronutrient deficiencies are corrected through single and multiple micronutrient supplements [40], lipid-based nutrient supplementation [40,41], food fortification, and provision of micronutrient powders [40]. Other studies have shown that lack of vitamin A supplementation is associated with higher odds of iron-deficiency anemia in LMIC in Africa, Asia, and the Caribbean [42] and that the protective effect of vitamin A supplementation on cognitive function and motor development is not evident in stunted children in Brazil [43].

The success of mega-dose vitamin A supplementation (200,000 IU) in the 1980s in reducing xerophthalmia [[44], [45], [46], [47], [48], [49], [50]] led to its adoption internationally. Subsequently, this intervention was not only shown to improve preschool children’s vitamin A status but also to improve linear growth and reduce diarrhea, respiratory, and gastrointestinal nematode infections [19,44,51]. These results led the Panamanian MoH to begin a mega-dose (200,000 IU) vitamin A supplementation program for preschool children in 2003 in the Ngäbe-Buglé comarca, an extremely impoverished indigenous region of western Panama. The MoH invited our team to determine if a mega-dose (200,000 IU) vitamin A supplement would reduce *Ascaris* transmission. We found that supplementation increased serum vitamin A concentrations from inadequate to adequate, that *Ascaris* reinfection following anthelmintic treatment was lower in nonstunted but not in stunted children after 3 mo, and that, after 5 mo, the benefit of the mega-dose supplement on *Ascaris* transmission in nonstunted children had waned [21]. Unpublished dietary data were also collected during this initial study and form the basis of the present study.

Our overarching goal was to integrate dietary variables with our database of maternal, sociodemographic, environmental, and infection-related variables in order to explore their relative impact on stunting in indigenous preschool children living in subsistence farming villages in rural Panama. Our specific objectives were as follows: *1*) to compare the prevalence of inadequate intakes of macro- and micronutrients between stunted and nonstunted preschool children; *2*) to compare dietary intake of specific foods, estimated intakes of macro- and micronutrients, incidence of diarrhea and respiratory infections, and prevalence and intensity of nematode infections between stunted and nonstunted preschool children during both the dry and rainy seasons; and *3*) to explore the relative contribution of sociodemographic factors, income, foods, estimated nutrient intakes, and infections in multivariate regression models for HAZ in each season.

## Methods

### Study site and population

In consultation with the Panamanian MoH, the present study was conducted on the outskirts of the *comarca* Ngäbe-Buglé, a geographically and administratively demarcated indigenous region in the Bocas Del Toro province, in the western, mountainous region near the Costa Rican border. According to national statistics at the time of data collection in 2003 [7], 90% of the population lived in poverty or extreme poverty. Houses were built on stilts to avoid flooding during the rainy season and to prevent the invasion of insects that nest and congregate in the grasses. Traditional houses had a single room with a cooking area at one end and a living space at the other. One or 2 sides were closed with walls of mud or wood, and roofs were made of thatch. Only 38% of the houses had some type of potable water supply, and 79% had latrines. There are 2 distinct seasons: a shorter dry season from mid-December to May, and a longer rainy season between May and December. Subsistence farming provided root vegetables, including cassava and yams, as well as rice, corn, beans, bananas, sugar, and coffee [52]. In similar Ngäbe-Buglé villages, it was reported that households categorized with the lowest index of wealth did not have an aqueduct, latrine, or stove, whereas those with a higher wealth index had a latrine and aqueduct, owned a radio, and may have had a cell phone, a sewing machine, and a stove [53].

In 2003, the *comarca* Ngäbe-Buglé had one of the worst health profiles in Panama, and the 5 leading causes of morbidity in preschool children treated at health posts were respiratory diseases, diarrhea and gastroenteritis, tuberculosis, malnutrition, and intestinal nematodes, including *Ascaris lumbricoides* and *Trichuris trichiura*. Compared to 2003, when there were only 1.42 nurses and 0.53 physicians for every 10,000 inhabitants in the *comarca* [7], in 2021, the number of physicians had increased to 1.39/10,000, whereas the number of nurses had declined to 1.1/10,000 [54]. Diarrhea remains a major cause of infant mortality in 2025 [55]. The MoH had begun a vitamin A supplementation program [200,000 IU (60 mg retinyl palmitate)] ∼6 mo prior to our study, but it had not yet reached all households. Albendazole treatment for intestinal nematode infections was also being administered every 6 mo, but none of the enrolled children had received anthelmintic treatment in the 4 mo prior to the beginning of the study. Stunting (61%) and *Ascaris* (80%) infection were very prevalent in this population of preschool children [21].

### Study design, recruitment, and relationship to our prior study

This was a prospective community-based study with data collection from the same cohort of children in the dry and rainy seasons. The ethics approval obtained from the institutional review board of McGill University (A12-M123-02B) was conditional on pre-approval from the Panamanian MoH through the Instituto Conmemorativo Gorgas de Estudios de la Salud, and from indigenous community leaders in Bocas del Toro.

Participants were recruited from February through April 2003 from 2 neighboring *corregimientos*, Risco and La Carretera, each consisting of 10‒15 villages with similar accessibility and poor health and nutritional status according to the MoH. A census conducted from February 2003 to April 2003 revealed a population of 790 children between 12 mo and 59 mo of age in our 24 study villages. The primary caregivers of 742 children (95% of the census population) were located and approached for participation in the study. After the exclusion of children who had a weight for height <‒2 SD below the mean, showed visible signs of vitamin A deficiency, had received a mega-dose (200,000 IU) vitamin A supplement between 3 mo and 6 mo prior to initiation of study, or had received an anthelmintic within the last 4 mo, 595 children were eligible for inclusion. Taking into account migration (*n* = 64), lack of follow-up stool samples (*n* = 71), and voluntary withdrawal from the study (*n* = 48), a complete data set was available for 328 children (41.5% of 790). Data collection was completed in 2003.

### Sociodemographic and living conditions

A standardized questionnaire administered to the primary caregiver during the dry season provided data on demographics (age and gender of children, breastfeeding duration, mega-dose vitamin A supplementation in the previous 3 mo), living conditions (individuals/household, type of floors, latrines, and household water supply), and household income in the previous month [21].

### Anthropometry

Child anthropometry was assessed during both the dry and rainy seasons from virtually all children (minimum 311) [56]. *Z*-scores for HAZ, weight-for-age (WAZ), and weight-for-height were calculated using EPIinfo, and HAZ, WAZ, and weight-for-height <‒2 SD were classified as stunted, underweight, and wasted, respectively.

### Infections

During both seasons, the primary caregiver reported the number of child episodes of diarrheal disease and respiratory infections in the previous month, chronic diseases, medications, and breastfeeding history. Fecal samples were collected and analyzed using the Kato-Katz technique for *Ascaris, Trichuris,* and hookworm prevalence (percentage infected) and intensity calculated as eggs per gram feces (epg) of feces [21]. Following the collection of the dry season sample, all children were dewormed with 400 mg albendazole in suspension form. The rainy season sample was collected 5 mo later.

### Food frequency and dietary diversity

A list of foods was developed during a pilot study in a nonparticipating community and used to create a semiquantitative food frequency questionnaire (FFQ), which was reviewed by key informants and health workers (Supplemental Table 1). The FFQ was then administered to the primary caregiver of a randomly selected nonbreastfeeding index child from each household once during both the dry (*n* = 162) and rainy (*n* = 190) seasons under the supervision of a dietitian. It provided data on the number of times foods had been consumed in the previous week. Based on the methodology of Torheim et al. [57], foods were categorized into 10 groups: dairy, fish, chicken, egg, meat, legumes, fruits, cereals, grains, and fats/oils. A diet diversity score was calculated for each child as the number of different food groups reported on the FFQ for the previous week. The total number of food servings per week was calculated as the sum of all servings from each of the 10 food groups.

### 24 h recall

A single 24 h dietary recall per season was administered on the day after the FFQ to age-stratified subgroups of 40 index children per age-group (12‒24 mo, 25‒36 mo, 37‒48 mo, 48‒60 mo) to record the number of servings on the previous day and the typical serving sizes. This smaller sample was necessary because of the remoteness of the communities, the distance between homes, and our limited personnel. This 24-h recall involved a multiple-pass interview. The first pass recorded when each food/beverage had been consumed and the preparation method. The records were reviewed during a second pass, and the quantity was determined using standard serving dishes and utensils. All results were reviewed during a third pass.

### Estimated serving sizes

The mean age-specific serving size was then calculated (grams per day) for each food on the FFQ [# weekly servings x mean serving size (g) / 7 d]. When recalls from the same children were available for both seasons (*n* = 150), the mean serving size per child was calculated. When recalls were available only from 1 season (dry season, 42; rainy season, 31), the single value was used.

### Estimated nutrient intakes

Age-specific nutrient analyses were then conducted using the database of Panamanian foods existing at the time of the study, together with FAO databases, to estimate daily intakes of 34 macronutrients, vitamins, and minerals for 223 children. Children with energy intakes <500 kcal or >3500 kcal (*n* = 6) were excluded from all dietary analyses. Daily estimated nutrient intakes were expressed per 1000 kcal.

### Adequacy of estimated nutrient intakes

Protein and energy requirements based on age, sex, and weight were calculated for each child using the values proposed by FAO/WHO joint consultations on requirements for energy [58], protein [59], and fat [60]. Energy and protein requirements were calculated based on minimum requirements recommended by WHO [58] of 90 kcal/kg for energy, 0.95 g/kg protein for children >36 mo, and 1.1 g/kg protein for children ≤36 mo of age. Targets for fat and saturated fat intake as a percentage of total energy intake for children >2 y were calculated using existing policy standards [61]. Recommended micronutrient requirements and the prevalence of inadequate intakes for vitamins and minerals were calculated using the cutoffs proposed by the FAO/WHO nutritional recommendations for micronutrients [62]. FAO/WHO-recommended nutrient intakes for zinc and iron for populations consuming foods with low bioavailability of these micronutrients were used [62].

### Statistical analysis

Statistical analysis was performed using Statistical Analysis System version 9.1 (SAS Institute Inc. 2002‒2003), and the level of significance was set at *P* < 0.05. For all variables, normality was diagnosed using the Shapiro-Wilk statistic. Differences between stunted and nonstunted children for continuous variables were identified using Wilcoxon Rank-Sum tests for nonnormally distributed data. As all *z*-scores were normally distributed, differences between stunted and nonstunted children were tested using analysis of variance (ANOVA) with age and income as covariates. The distributions of nematode intensities were normalized using the natural log transformation. The χ^2^ tests were used to compare stunted with nonstunted children for categorical variables (percentage female, type of flooring in the home, access to a latrine, and prevalence of underweight, wasting, and *Ascaris* and *Trichuris* ln epg).

Nonparametric statistics were used to test differences in food group intake between stunted and nonstunted children. Within each season, the mean number of weekly servings from each food group, total number of food servings per week, and dietary diversity scores were compared using Friedman’s 2-way ANOVA, adjusting for age. Friedman’s nonparametric repeated measure ANOVA was used to examine differences between the dry and rainy seasons in mean weekly number of servings from each food group, total number of food servings per week, and dietary diversity score*s* for all children. Differences in usual daily macronutrient and micronutrient intakes between stunted and nonstunted children were examined using ANOVA, controlling for age and income covariates. The percent of stunted and nonstunted children meeting recommended requirements for macronutrients and micronutrients was compared using χ^2^ tests.

An iterative process of stepwise linear regression was used to establish models that predicted HAZ in the dry and rainy seasons using the following independent variables: age, sex, monthly household income, mother’s education, access to a latrine, dirt floors in the home, as well as season-specific ln *Ascaris* epg, ln *Trichuris* epg, number of episodes of diarrhea and respiratory infections in the previous month, and either food group or usual nutrient intakes. To ensure the assumption of normality was met for each model, the distributions of the residuals were plotted and ascertained using kernel density estimates, quantile-quantile plots, and the Shapiro-Wilk statistic. The variance inflation factor was used to indicate multicollinearity, and values <10 were considered acceptable. For all models, only independent variables that entered the model with a *P* value <0.15 were included in the final model, with the exception of usual daily energy intake, which was forced into all final models and retained even when nonsignificant.

## Results

### Population and infection characteristics by season and stunting

Basic sociodemographic characteristics (Table 1) revealed a moderately high percentage of households with access to a household water supply (71.2%) and a latrine (72.7%), and only a few homes with dirt floors (7.3%). Stunting was detected in 61% of children in the dry season and 64.3% in the rainy season. Stunted children were older than nonstunted children, and stunted index children came from households with a monthly income approximately half that of households with nonstunted index children (38.5 United States dollars compared with 72 United States dollars per month, respectively). At the time of the study, 62% of nonstunted and 59% of stunted children had received a vitamin A supplement within the previous 3 mo.

**TABLE 1:** Individual and household characteristics of stunted compared with nonstunted children at the first interview (dry season).^1^

	Nonstunted	Stunted
Individual		
Number of children	128	200
Age, month	33 (1)^a^	37 (1)^b^
Female, %	52	45
Breastfeeding duration, month^2^	16.5 (0.8)	16.6 (0.6)
Vitamin A supplement in the previous 3 mo, %	62	59
Household		
Number of households^3^	83	122
Mother’s education, y	4.4 (0.3)	3.8 (0.2)
Household income, United States dollar/month	72.0 (10.3)^b^	38.5 (4.7)^a^
Individuals/household	9.8 (0.5)	10.1 (0.5)
Dirt floors in the home, %^4^	6 (1‒11)	9 (4‒14)
Latrine access, %	78 (69‒87)	70 (62‒78)
Household water supply, %^5^	76 (67‒85)	70 (62‒78)

Abbreviations: CI, confidence interval; HAZ, height-for-age *z*-score; SE, standard error.

1 Values are mean (SE) or percent (95% CI). Different letter superscripts indicate significant differences using Wilcoxon’s Rank-Sum or χ^2^ tests (*P* < 0.05).

2 Included only children who had completed breastfeeding (nonstunted: *n* = 98; stunted: *n* = 164).

3 Households defined as stunted/nonstunted based on the HAZ of the index child at the initial interview.

4 Others had wood or cement floors.

5 Others used wells or rivers as a water source.

Health indicators (Table 2) showed that the WAZ score was lower in stunted than nonstunted children and that none of the children were underweight or wasted. Diarrhea was more common than respiratory infections, and the number of diarrhea episodes was higher during the rainy season (dry: 1.66/mo; rainy: 2.25/mo). *Ascaris* prevalence (dry: 80.8%; rainy: 65.9%) was higher than *Trichuris* (dry: 11.6%; rainy: 11.3%). Furthermore, *Ascaris* prevalence was higher in stunted than nonstunted children in both dry and rainy seasons, and *Ascaris* intensity was higher in stunted children only in the rainy season (Table 2). In contrast, respiratory infections were more common in nonstunted than stunted children and only in the dry season.

**TABLE 2:** Comparison of *z*-scores for weight for age and weight for height, gastrointestinal nematodes, and diarrheal and respiratory infections between nonstunted and stunted children in the dry and rainy seasons.^1^

	Dry season		Rainy season	
	Nonstunted (*n* = 128)	Stunted (*n* = 200)	Nonstunted (*n* = 117)	Stunted (*n* = 211)
Mean WAZ	‒0.3 (0.1)^a^	‒1.3 (0.1)^b^	‒0.1 (0.1)^a^	‒1.2 (0.1)^b^
Mean WHZ	0.7 (0.8)	0.6 (0.1)	0.7 (0.1)	0.7 (0.1)
GI nematode infection				
*Ascaris* prevalence, %	73 (65‒80)^a^	86 (81‒91)^b^	55 (46‒64)^a^	72 (66‒78)^b^
*Ascaris* intensity, epg	19,147 (3801)	22,951 (3323)	7914 (1737)^a^	8906 (853)^b^
*Trichuris* prevalence, %	18 (11‒25)	20 (14‒26)	17 (10‒24)	20 (15‒25)
*Trichuris* intensity, epg	57 (19)	131 (73)	86 (40)	56 (19)
Other infections (# in previous month)				
Diarrheal episodes	1.9 (0.3)	1.5 (0.2)	1.8 (0.2)	2.5 (0.2)
Respiratory infections	1.4 (0.1)**^b^**	1.2 (0.1)^a^	0.7 (0.1)	0.9 (0.1)

Abbreviations: ANOVA, analysis of variance; CI, confidence interval; epg, eggs per gram feces; GI nematode, gastrointestinal nematode; SE, standard error; WAZ, weight-for-age *z*-score; WHZ, weight-for-height *z-*score.

1 Values are mean (SE) or percent (95% CI). Different letter superscripts indicate significant differences between stunted and nonstunted children within each season using Wilcoxon’s Rank-Sum test, ANOVA with age and income as covariates, or χ^2^ tests (*P* < 0.05).

### Food group and nutrient intakes

Child diet diversity did not differ by season, but children had more food servings per week in the dry season than in the rainy season (Table 3). Children consumed less animal-source foods (dairy, eggs), vegetables, and cereals/grains during the rainy season, whereas weekly servings of fats/oils were lower in the dry season compared to the rainy season.

**TABLE 3:** Food servings, diet diversity, and weekly food group intakes of stunted compared with nonstunted children in each season based on food frequency questionnaires at each season.^1^^,^^2^

Foods	Dry season			Rainy season		
	Total (*n* =162)	Nonstunted (*n* = 68)	Stunted (*n* = 94)	Total (*n* = 190)	Nonstunted (*n* = 68)	Stunted (*n* = 122)
Dietary diversity	7.2 (0.1)	7.4 (0.2)	7.1 (0.2)	7.0 (0.1)	7.3 (0.2)	6.8 (0.2)
Total servings/wk, *n*	63.3 (2.7)^A^	62.5 (3.3)	63.9 (3.9)	51.6 (1.4)^B^	52.6 (2.1)	51.1 (1.8)
Animal source	13.3 (0.9)^A^	15.1 (1.2)^a^	12.0 (1.2)^b^	9.2 (0.6)^B^	10.7 (1.1)^a^	8.3 (0.7)^b^
Dairy	5.4 (0.7)^A^	7.3 (1.0)^a^	4.0 (0.8)^b^	2.7 (0.4)^B^	3.8 (0.8)	2.0 (0.5)
Fish	1.6 (0.2)	1.6 (0.3)	1.5 (0.2)	1.4 (0.1)	1.5(0.2)	1.3 (0.2)
Chicken	2.2 (0.2)	2.3 (0.3)	2.1 (0.2)	2.0 (0.2)	2.4 (0.2)	1.8 (0.2)
Egg	3.1 (0.3)^A^	2.9 (0.4)	3.3 (0.4)	2.1 (0.2)^B^	2.5 (0.3)	1.9 (0.2)
Meat	1.1 (0.1)	1.0 (0.1)	1.1 (0.1)	0.9 (0.1)	0.9 (0.2)	0.9 (0.1)
Vegetables	10.7 (0.9)^A^	7.8 (0.9)^b^	12.8 (1.4)^a^	6.8 (0.4)^B^	7.1 (0.9)	6.7 (0.5)
Roots and tubers	6.6 (0.7)^A^	4.7 (0.7)^b^	7.9 (1.0)^a^	3.5 (0.3)^B^	3.2 (0.7)^b^	3.7 (0.4)^a^
Plantain	3.2 (0.3)	2.5 (0.3)	3.7 (0.5)	2.4 (0.2)	3.0 (0.4)	2. 1 (0.2)
Fruits	16.8 (0.9)	16.7 (1.4)	16.8 (1.1)	15.4 (0.5)	13.9 (0.9)^b^	16.2 (0.6)^a^
Banana	10.6 (0.6)	11.2 (1.0)	10.1 (0.7)	10.1 (0.4)	9.2 (0.6)	10.6 (0.5)
Pifa^3^	4.1 (0.5)	3.5 (0.7)	4.6 (0.6)	2.6 (0.2)	2.6 (0.3)^b^	3.1 (0.3)^a^
Cereals/grains	8.0 (0.6)^A^	8.2 (0.8)	7.9 (0.8)	5.7 (0.3)^B^	7.0 (0.6)^a^	4.9 (0.4)^b^
Rice	6.5 (0.5)	6.5 (0.7)	6.5 (0.7)	4.7 (0.3)	5.7 (0.5)^a^	4.1 (0.3)^b^
Bread	1.2 (0.2)	1.3 (0.2)	1.1 (0.2)	0.8 (0.1)	1.1 (0.2)^a^	0.7 (0.1)^b^
Tortilla	0.3 (0.1)	0.4 (0.2)	0.3 (0.1)	0.1 (0.05)	0.1 (0.06)	0.1 (0.06)
Legumes	2.3 (0.3)	3.2 (0.6)^a^	1.8 (0.3)^b^	1.9 (0.2)	2.3 (0.3)^a^	1.6 (0.3)^b^
Fats/oils	1.3 (0.2)^B^	1.9 (0.3)	0.9 (0.2)	2.2 (0.2)^A^	2.8 (0.4)^a^	1.8 (0.2)^b^

Abbreviations: ANOVA, analysis of variance; SE, standard error.

1 Data reported as mean number of servings per week (SE).

2 Different uppercase superscripts indicate differences between the total sample in dry compared with rainy seasons, using Friedman’s nonparametric equivalent of a paired t-test (*P* < 0.05). Different lowercase superscripts indicate significant differences between stunted and nonstunted children when controlling for age and sex using Friedman’s nonparametric ANOVA.

3 Pifa (peach palm) is a local fruit tree.

Diet differed between stunted and nonstunted children (Table 3). Stunted children in both seasons had fewer servings of animal-source foods and more servings of roots and tubers, albeit significantly lower in the rainy season. Additionally, during the rainy season, stunted children consumed fewer servings of cereals/grains and fats/oils, but more servings of fruits, specifically *pifa* (a peach palm fruit), than nonstunted children.

Total carbohydrate, total sugar, and dietary fiber intakes were higher among stunted children, whereas intakes of total fat and of both MUFA and PUFA were lower in stunted children (Table 4 [[45], [46], [47], [48]]). The percentage of children not meeting total fat requirements was higher for stunted compared with nonstunted children (Table 4). Fat intakes were exceptionally low, with preschool children consuming only 14‒16% of their total energy intake as dietary fat compared with the recommendation of 25‒40%, and <1% for both MUFA and PUFA, compared with recommendations of 13% for MUFA and 6.5% PUFA.

**TABLE 4:** Energy-adjusted estimated daily nutrient intakes and prevalence of inadequacy in stunted and nonstunted children based on 2 semiquantitative food frequency questionnaires.^1^

Nutrients	Estimated intakes^2^		% Not meeting requirements^3^	
	Nonstunted (*n* = 85)	Stunted (*n* = 132)	Nonstunted (*n* = 85)	Stunted (*n* = 132)
Energy, kcal	1680 (69)	1634 (46)	15	11
Macronutrients (/1000 kcal)				
Protein, g	27.2 (0.7)	26.9 (0.6)	4	7
Carbohydrates, g	176 (3)^b^	188 (3)^a^	--	--
Total sugar, g	74.1 (1.8)^b^	82.8 (1.6)^a^	--	--
Dietary fiber, g	20.8 (1.0)^b^	24.2 (0.8)^a^	--	--
Total fat, g	30.0 (1.4)^a^	26.4 (1.0)^b^	52^b^	70^a^
Sat fat, g	7.7 (0.2)	7.4 (0.2)	--	--
MUFA, g	8.4 (0.3)^a^	7.4 (0.3)^b^	--	--
PUFA, g	11.8 (0.9)^a^	9.5 (0.7)^b^	--	--
Cholesterol, mg	80.9 (5.5)	67.8(3.4)	--	--
Vitamins (/1000kcal)				
Total vitamin A, RAE	178 (10)	212 (13)	79	73
Retinol, *μ*g	83.8 (7.2)^a^	66.1 (5.6)^b^	--	--
β-carotene, *μ*g	1004 (110)	1629 (145)	--	--
α-carotene, *μ*g	248 (16)	244 (12)	--	--
Cryptoxanthin, *μ*g	36.7 (4.4)	34.1 (3.3)	--	--
Lutein/zeaxanthin, *μ*g	2224 (343)^b^	4196 (444)^a^	--	--
Lycopene, *μ*g	1.27 (0.7)	0.48 (0.14)	--	--
α-tocopherol, *μ*g	4.2 (0.2)	4.1 (0.2)	38	47
Thiamin, mg	0.46 (0.01)	0.45 (0.01)	35	33
Riboflavin, mg	0.69 (0.02)	0.71 (0.01)	11	9
Niacin, mg	6.8 (0.1)	7.0 (0.1)	15	15
Vitamin B_6_, mg	1.66 (0.05)	1.88 (0.05)	10	10
Folate, DFE	162 (5)	159 (3)	25	20
Vitamin B_12_, mg	0.88 (0.06)	0.77 (0.04)	43^b^	62^a^
Vitamin C, mg	54.8 (2.1)^b^	61.9 (1.8)^a^	12	9
Minerals (/1000 kcal)				
Calcium, mg	254 (12)	245 (10)	77	80
Phosphorus, *μ*g	582 (22)	603 (18)	--	--
Magnesium, mg	298 (16)^b^	349 (13)^a^	3	4
Iron, mg	7.8 (0.4)	8.6 (0.4)	62	56
Zinc, mg	5.2 (0.2)	5.5 (0.2)	62	64
Copper, mg	1.6 (0.1)^b^	1.9 (0.1)^a^	--	--
Selenium, *μ*g	32.8 (0.9)	30.8 (0.7)	5	8
Potassium, mg	2566 (87)^b^	2940 (77)^a^	--	--
Sodium, mg	2302 (139)	2048 (198)	--	--

Abbreviations: CI, confidence interval; DFE, dietary folate equivalents; FAO, Food and Agriculture Organization; MUFA, monounsaturated fatty acid; PUFA, polyunsaturated fatty acid; RAE, retinoic acid equivalents; SE, standard error; WHO, World Health Organization; DFE; RAE.

--indicates no FAO/WHO proposed cutoff at time of study.

1 Data presented as the mean (SE) or as percent (95% CI). Different letter superscripts indicate differences in mean intake or prevalence of inadequacy between stunted and nonstunted children.

2 Usual nutrient intake based on average of 2 24 h recalls (1 per season, *n* = 150) or a single 24 h recall (*n* = 77).

3 Energy requirements [46], protein requirements [47], fat requirements [48], micronutrient requirements based on recommendations for populations with a low bioavailability of nutrients from dietary sources [45].

There was a high overall prevalence of inadequacy of micronutrients in both stunted and nonstunted children (Table 4), particularly for vitamin A, calcium, zinc, and iron, where >60% children did not meet requirements. In addition, the prevalence of inadequate vitamin B_12_ intake was significantly higher in stunted children (62% compared with 43%). Retinol intake was also lower in stunted children, although intake of plant sources of vitamin A (lutein/zeaxanthin) was higher in stunted children. Consistent with higher fruit intakes, daily vitamin C intake was higher in the stunted children. Intakes of magnesium, copper, and potassium were also higher in stunted children.

### Predictors of HAZ

Multiple linear regression analyses were performed to determine the independent predictors of HAZ during both the dry and rainy seasons using food group (Table 5) and nutrient (Table 6) models. In the food group models, the dry season model accounted for 25% of the observed variability in HAZ and revealed that female, income, and weekly intake of fats/oils were positively associated with HAZ, and that older age was negatively associated with HAZ. The rainy season model accounted for 31% of the variability in HAZ. As during the dry season, age and weekly intake of fats/oils were negatively associated with HAZ. In addition, HAZ was positively associated with weekly servings of grains/cereals and negatively associated with fruit servings. Of note, a highly significant negative association of HAZ with the monthly incidence of diarrheal episodes was detected, but only in the rainy season.

**TABLE 5:** Demographic, socioeconomic, food group, and infection predictors of height-for-age *z*-score in each season through stepwise linear regression.^1^

Independent variables	Dry season		VIF	Rainy season		VIF
	β (SE)	*P*		β (SE)	*P*	
Age, mo	‒0.02 (0.01)	0.0002	1.09	‒0.02 (0.006)	0.001	1.10
Female	0.33 (0.17)	0.05	1.02	NE	-	-
Socioeconomic status/living conditions^2^						
Income, United States dollars	0.004 (0.001)	0.003	1.01	-	-	-
Individuals/household	‒0.03 (0.02)	0.11	1.03	‒0.03 (0.02)	0.08	1.16
Dirt floors in the home	NE	-	-	NE	-	-
Weekly food group intake^3^						
Meat	NE	-	-	‒0.1 (0.1)	0.12	1.10
Legumes	NE	-	-	NE	-	-
Tubers	‒0.02 (0.01)	0.08	1.11	NE	-	-
Fruits	NE	-	-	‒0.04 (0.01)	0.002	1.19
Grains/cereals	NE	-	-	0.05 (0.02)	0.01	1.33
Fats/oils	0.12 (0.04)	0.003	1.10	0.08 (0.04)	0.04	1.78
Usual nutrient intake						
Energy, kcal	1 x 10^‒4^ (2 x 10^‒4^)	0.50	1.21	‒1 x 10^‒4^ (2 x 10^‒4^)	0.59	1.84
Illness/nematode infection^3^						
Diarrheal episode /mo^4^	NE	-	-	‒0.09 (0.03)	0.0007	1.14
Intercept	‒1.58 (0.37)	<0.0001	0	‒0.87 (0.37)	0.02	0
_R_2	0.2482			0.3124		
F value	6.27			8.13		
*P* value	<0.0001			<0.0001		
*N*	161			171		

Abbreviations: epg, eggs per gram feces; NE, not entered; SE, standard error; VIF, variance inflation factor.

1 Variables selected using stepwise linear regression. Final models included all variables with a *P* value <0.15 and were adjusted for total energy intake. Variables included in models that did not enter in either season: weekly consumption of dairy, egg, fish, chicken; *Ascaris* ln epg, *Trichuris* ln epg; dirt floors in home; mother’s education.

2 Measured only in the dry season.

3 Measured in both seasons. Neither *Ascaris* nor *Trichuris* infection entered the models.

4 Measured in the previous month.

**TABLE 6:** Demographic, socioeconomic, specific nutrient and infection predictors of height-for-age *z*-score.^1^

Independent variables	Dry season		VIF	Rainy season		VIF
	β (SE)	*P*		β (SE)	*P*	
Age, mo	‒0.02 (0.01)	0.001	1.10	‒0.02 (0.006)	0.0003	1.11
Female, (1 = yes; 0 = no)	0.27 (0.15)	0.08	1.04	NE	-	-
Socioeconomic status/living conditions^2^						
Income, United States dollars	0.003 (0.001)	0.03	1.12	0.002 (0.001)	0.12	1.13
Individuals/household, *n*	‒0.03 (0.02)	0.05	1.03	‒0.02 (0.01)	0.10	1.05
Dirt floor, (1 = yes; 0 = no)	‒0.5 (0.3)	0.05	1.04	‒0.6 (0.3)	0.03	1.07
Usual nutrient intake						
Energy, kcal	8 x 10^‒5^ (1 x 10^‒4^)	0.62	1.15	9 x 10^‒5^ (1 x 10^‒4^)	0.51	1.12
Total sugar, g/1000 kcal	‒0.01 (0.01)	0.009	1.24	‒0.01 (0.01)	0.001	1.22
Illness/nematode infection^3^						
*Ascaris* ln (epg)	NE	-	-	‒0.03(0.02)	0.13	1.13
Diarrheal episodes/mo^4^	NE	-	-	‒0.12 (0.03)	<0.0001	1.07
Intercept	‒0.66 (0.5)	0.19	0	‒0.02 (0.49)	0.96	0
R^2^	0.1921			0.2682		
F value	6.86			8.57		
*P* value	<0.0001			<0.0001		
*N*	209			196		

Abbreviations: epg, eggs per gram feces; NE, not entered; SE, standard error; VIF, variance inflation factor.

1 Variables selected using stepwise linear regression. Final models included all variables with *P* value <0.15 and were adjusted for total energy intake. Variables included in the models that did not enter in either season: mother’s education, intakes per 1000 kcal of protein, total fat, dietary fiber, retinol, lutein/zeaxanthin, vitamin C, riboflavin, magnesium, copper, potassium; *Trichuris* ln epg.

2 Only measured in the dry season.

3 Measured in both seasons.

4 Measured in previous month.

When models included specific nutrients (Table 6) instead of food groups, the multiple linear regression model during the dry season accounted for 19% of variability in HAZ, and showed that income was positively associated with HAZ whereas older age, household crowding, presence of a dirt floor, and higher grams of total sugar per 1000 kcal were negatively associated with preschool child HAZ. The rainy season nutrient model captured 27% of the variability in HAZ, and household income was not significant. Negative associations with HAZ were found for age, dirt floors, and higher total grams of sugar. Diarrheal episodes in the previous month entered as a highly significant negative association with HAZ in the nutrient model in the rainy season, as was observed in the food model.

## Discussion

In Latin America, both undernutrition and micronutrient deficiencies are considered public health concerns [63]. This study allowed us to explore the relative contribution of nutritional, infection, and sociodemographic variables to low HAZ in an extremely impoverished rural indigenous Panamanian population where over 60% of preschool children were stunted. We observed that diarrhea and *Ascaris* infections were common, and that diets were not only severely limited in fats/oils and high in sugar but also provided inadequate intakes of multiple micronutrients, where the prevalence of inadequacy exceeded 50% for vitamin A, vitamin B_12_, calcium, iron, and zinc. In this context, HAZ was positively associated with intakes of fats/oils and negatively associated with sugar in both the dry and rainy seasons. Moreover, diarrheal episodes had a strong negative association with HAZ in both food and estimated nutrient intake models, but only during the rainy season. In the dry season, income entered both models as positively associated with HAZ. Collectively, these findings show that linear growth faltering in preschool children is multifactorial and that improvements in diet quality and reduction of diarrhea will likely improve linear growth.

### Infections

The lower prevalence of *Ascaris* in the rainy season compared with the dry season was expected because albendazole treatment during the dry season would have expelled close to 100% of adult worms [64]. Reinfection occurs through ongoing ingestion of eggs in the environment, increasing the prevalence to pretreatment levels more rapidly than intensity [65]. Consistent with other studies [18,23,66,67], *Ascaris* was associated with stunting. In the rainy season, stunted children not only had higher reinfection intensity than nonstunted children but also had higher intakes of fruits and roots/tubers. *Ascaris* eggs stick to the surface of unwashed fruits and vegetables [27,64], and thus the higher consumption of these potential sources of infection could have contributed to the higher *Ascaris* intensity in stunted children in the rainy season. Given the relatively high prevalence of *Ascaris* in the present study, the *Ascaris*-induced malabsorption of fat and vitamin A [19,20,38,68] combined with the observed low dietary intakes of fat would have further reduced net fat absorption in these children. Despite this, however, *Ascaris* did not emerge as a significant determinant of HAZ in models that included both incidence of diarrhea and foods or estimated nutrient intakes.

In contrast to *Ascaris*, the incidence of diarrhea had a strong negative association with HAZ in both the food and nutrient models in the rainy season. This is consistent with many epidemiological studies that link diarrhea with stunting or low HAZ [5,69,70]. A meta-analysis of 9 studies from Peru and Brazil [69] revealed that a history of diarrhea was associated with stunting in 2-y-olds. Furthermore, a secondary analysis of meta-analyses between 1970 and 2015 in Latin America, Asia, and Africa reported that diarrhea explained the highest population attributable fraction of stunting (0.25), compared with other infections (hookworm, HIV), diet diversity, feeding frequency, low birthweight, and pollutant biomass [29]. Despite these studies on stunting and diarrhea, to our knowledge, ours is the first to include episodes of diarrhea along with foods or estimated nutrient intakes in multiple regression models of HAZ. Remarkably, diarrhea emerged as the most significant determinant of HAZ in both the food and the nutrient models in the rainy season. This observed negative association with HAZ is consistent with the impaired absorption of nutrients that occurs during diarrhea [30,71]. Moreover, given that zinc supplementation has been shown to have prophylactic effects on diarrhea [[72], [73], [74]] and that dietary intake of zinc was inadequate in the majority of children in this study, a zinc supplementation program would be beneficial in reducing diarrhea during the rainy season.

### Unbalanced macronutrient intakes

Low protein intakes are generally associated with growth faltering [75], yet, despite the high rate of stunting in this population, almost all children had adequate intakes of protein. However, we uncovered seasonal differences in intakes of protein-source foods that were associated with stunting; servings of cereal/grains were lower in the rainy compared to the dry season, and were also lower in stunted compared to nonstunted children in the rainy season. In addition, stunted preschool children had fewer weekly servings of dairy and eggs in the dry season and of legumes in both seasons. Our finding that nonstunted children consumed more dairy aligns with earlier observations that consumption of milk, but not fish, chicken, eggs, or meat, was associated with higher HAZ [76] and with evidence that dairy is a source of insulin growth factor-1 [77]. Furthermore, neither animal-source proteins nor legumes entered our HAZ food model, and protein did not enter the HAZ nutrient model in either season. Cereals/grains, but not meat, were a significant and positive determinant of HAZ only in the rainy season. This supports a review of intervention studies on preschool children from LMIC countries that noted little difference between meat compared with cereal-based intakes on HAZ [78]. Given the adequate intakes of protein in these children, protein deficiency is unlikely to contribute to stunting.

In contrast to adequate protein intakes, inadequate intakes of fat were observed in both nonstunted (52%) and stunted (70%) preschool children. Early reports had identified growth faltering in children under 6 y when the diet provided <22% of energy from fat [79] and when fat intakes were <12% of total energy intake in Mexico [80,81]. Currently, there is growing concern about low dietary fat and essential fatty acid intakes internationally [82] as well as in Latin America [83]. Low fat intakes lead to deficiencies of essential fatty acids and fat-soluble vitamins D and E, which are common in Latin America [84]. Recent guidelines for children >2 y of age recommend that 25‒40% of total energy intake should come from fat [61]. However, children in our study obtained only 14‒16% of their energy from fat, and children with higher fat intake had higher HAZ. Moreover, compared with recommendations for MUFA of 13% of total energy and for PUFA of 6.5% of total energy [61], these indigenous Panamanian preschool children consumed <1% of both MUFA and PUFA. Recently, stunting was associated with low blood concentrations of essential fatty acids in children <5 in LMIC, even in the absence of protein-energy malnutrition [85], reinforcing concerns about the association of essential fatty acid deficiencies with stunting in preschool children.

Preschool children consumed a variety of carbohydrates, including roots and tubers, plantain, banana, pifa, rice, bread, tortilla, as well as legumes. Of note, however, was the high intake of total sugar, which amounted to nearly 20% of total energy intake, far exceeding WHO-recommended intakes of <5% [86]. In addition to sugars in fruits and vegetables, the main dietary source was cane sugar added to coffee. In Latin America, it is a common practice for mothers to provide diluted coffee sweetened with sugar to allay children’s hunger when food is limited [87]. This practice, which contributes few micronutrients but increases energy intake, has been associated with stunting in Guatemala [87]. In our study, total sugar was inversely associated with HAZ in preschool children in both seasons, demonstrating that sugar provided only “empty calories” with no essential micronutrients. Moreover, the negative association of fruits with HAZ in the rainy season might be explained by the low fat and essential fatty acid content of fruits, together with their high sugar content. Given the high sugar intake together with low fat intake, our data strongly support lowering the sugar intake in preschool children’s diet and increasing fat intake to rebalance the intake of macronutrients and thus promote linear growth.

### High prevalence of micronutrient inadequacies

A range of micronutrient deficiencies remains a common problem in Latin America [88,89]. In our study, the prevalence of inadequate micronutrient intakes was very high in preschool children with inadequate intakes of vitamin A and calcium exceeding 75%, iron and zinc exceeding 55% as well as vitamin B_12_. In impoverished South African preschoolers, low intakes of calcium and vitamin D and vitamin E were common, and low fat, calcium, vitamin D, and vitamin B_12_ were observed in stunted children [90]; others have identified similar micronutrient deficiencies in Malaysia toddlers despite adequate protein intakes [91]. In our study, even in the presence of adequate protein intake, intakes of vitamin B_12_ were low, and the prevalence of inadequate intakes of vitamin B_12_ was higher in stunted (62%) than nonstunted (43%) preschool children. Although vitamin B_12_ intake is often tied to animal-source foods, a study of Ngäbe-Buglé preschool children reported that serum vitamin B_12_ was positively associated with servings of fruit, corn-based foods, traditional starchy foods including yam (ñame), and negatively associated with pigeon peas (guandú) as well as diarrhea [92].

Inadequacy of calcium intake was extremely common (>75%), and daily calcium intake averaged only 250 g/1000 kcal/d (equivalent to 400 g/d). In order to sustain linear growth, not only is ongoing intake of dietary calcium necessary for bone remodeling in preschool children [93], but also ≥700 mg calcium per day is needed for linear growth [94], a value considerably higher than the intake observed in the present study. However, even though nonstunted children had more dairy servings per week in the dry season, neither dairy servings nor calcium intake entered the HAZ models. Furthermore, dairy servings were even lower in the rainy season. Thus, strategies to increase calcium intake in indigenous preschool children are needed.

In Latin America, mega-dose supplementation with vitamin A as well as iron is typically used for toddlers and preschool children [89]. Only 2 studies have investigated the combination of mega-dose vitamin A supplementation with dietary interventions [49,95]. Pant et al. [49] showed that combining 200,000 IU of vitamin A with training mothers to identify vitamin A-rich foods increased the risk reduction for xerophthalmia in Nepalese children. Sheftel et al. [95] highlighted the risk of hypervitaminosis A and lower anthropometric indices in South Africa when mega-dose vitamin A accompanied diets that included vitamin A fortified foods, as well as sheep’s liver, which provides adequate vitamin A [96]. Hence, caution is needed when administering mega-dose vitamin A to populations with adequate dietary vitamin A intakes.

### Strengths and limitations

Strengths of this study were the multivariable data set collected during 2 seasons and the high coverage (41.5%) of preschool children from difficult-to-reach, impoverished indigenous villages, where stunting but not wasting was prevalent. Together, our findings provide relevant insights for extremely impoverished, remote indigenous populations that still persist today. However, we acknowledge the following limitations. It was not possible to obtain 24 h recalls from all children in both seasons. Episodes of diarrhea, respiratory infections, food frequency, and 24 h recalls were based on the mother’s recall. The study duration included only 1 dry and 1 rainy season. As with any observational study, we were unable to establish causality.

In conclusion, in this rural indigenous community where over 60% of preschool children were stunted and diarrhea and *Ascaris* infections were common, diets were severely limited in fats/oils and high in sugar. This unhealthy macronutrient distribution would explain the high prevalence of inadequate intakes of essential fatty acids, fat-soluble vitamins, and multiple micronutrients. A shift from a high proportion of energy from sugar to fats and oils with more micronutrient-rich foods would improve diet quality and HAZ in preschool children. Efforts to lower the incidence of diarrhea would also benefit linear given its strong negative association with HAZ in these preschool children during the rainy season. Taken together, these findings show that linear growth faltering in impoverished, remote preschool children is multifactorial and that improvements in diet quality and reduction of diarrhea are likely to improve linear growth.

## Author contributions

The authors’ responsibilities were as follows. – EO-B: established partnerships with the Ministry of Health and indigenous community leaders; DIK, KGK, MES, EO-B: designed the study; DIK, MES, KGK: analyzed data; KGK, MES, DIK: wrote the article; KGK, MES: had primary responsibility; and all authors: read and approved the final manuscript.

## Funding

Fieldwork was supported by a Canadian Institute for Health Research Global Health Planning and Pilot Grant (GLH-64075) to KGK. Laboratory and data analyses were supported by Natural Sciences and Engineering Research Council of Canada Discovery Grants A3585 (MES) and A3623 (KGK). These supporting sources had no restrictions regarding the publication of the work.

## Conflict of interest

The authors report no conflicts of interest.

## References

[1] Solomons N.W., Vossenaar M., Chomat A.M., Doak C.M., Koski K.G., Scott M.E. (2014). Stunting at birth: recognition of early-life linear growth failure in the western highlands of Guatemala, Publ. Health Nutr.

[2] González-Fernández D., Nemeth E., Pons E.D., Rueda D., Sinisterra O.T., Murillo E. (2021). INTERGROWTH-21 identifies high prevalence of low symphysis–fundal height in indigenous pregnant women experiencing multiple infections, nutrient deficiencies, and inflammation: the maternal infections, nutrient deficiencies, and inflammation (MINDI) cohort. Curr. Dev. Nutr..

[3] United Nations (2019). https://sdgs.un.org/goals/goal2.

[4] Lee B.X., Kjaerulf F., Turner S., Cohen L., Donnelly P.D., Muggah R. (2016). Transforming our world: implementing the 2030 agenda through Sustainable Development Goal indicators. J Public Health Policy.

[5] Gausman J., Kim R., Li Z., Tu L., Rajpal S., Joe W. (2022). Comparison of child undernutrition anthropometric indicators across 56 low- and middle-income countries. JAMA Netw. Open.

[6] de Onis M., Frongillo E.A., Blössner M. (2000). Is malnutrition declining? An analysis of changes in levels of child malnutrition since 1980. Bull. World Health Organ..

[7] Ministerio de Salud (2003).

[8] Matos S.M., Amorim L.D., Campos A.C., Barreto M.L., Rodrigues L.C., Morejón Y.A. (2017). Growth patterns in early childhood: better trajectories in Afro-Ecuadorians independent of sex and socioeconomic factors. Nutr. Res..

[9] Gatica-Domínguez G., Victora C., Barros A.J. (2019). Ethnic inequalities and trends in stunting prevalence among Guatemalan children: an analysis using national health surveys 1995–2014. Int. J Equity Health.

[10] Ghattas H., Acharya Y., Jamaluddine Z., Assi M., El Asmar K.E., Jones A.D. (2020). Child-level double burden of malnutrition in the MENA and LAC regions: prevalence and social determinants, Matern. Child Nutr..

[11] Ervin P.A., Bubak V. (2019). Closing the rural-urban gap in child malnutrition: evidence from Paraguay, 1997–2012. Econ. Hum. Biol..

[12] Takele K., Zewotir T., Ndanguza D. (2019). Understanding correlates of child stunting in Ethiopia using generalized linear mixed models. BMC Public Health.

[13] Rivera A., Marín V., Romaní F. (2024). Concurrence of anemia and stunting and associated factors among children aged 6 to 59 months in Peru. PLOS Glob. Public Health..

[14] Susiloretni K.A., Smith E.R., Suparmi A.R., Marsum R.A., Agustina R., Shankar A.H. (2021). The psychological distress of parents is associated with reduced linear growth of children: evidence from a nationwide population survey. PLOS One.

[15] Zavala E., King S.E., Sawadogo-Lewis T., Roberton T. (2021). Leveraging water, sanitation and hygiene for nutrition in low- and middle-income countries: a conceptual framework. Matern. Child Nutr..

[16] Agabiirwe C.N., Dambach P., Methula T.C., Phalkey R.K. (2022). Impact of floods on undernutrition among children under five years of age in low- and middle-income countries: a systematic review. Environ. Health..

[17] Huicho L., Vidal-Cárdenas E., Akseer N., Brar S., Conway K., Islam M. (2020). Drivers of stunting reduction in Peru: a country case study. Am. J Clin. Nutr..

[18] Loglo A., Aniagyei W., Vivekanandan M.M., Agbanyo A., Asamoah E.A., Phillips R.O. (2024). A systematic review and meta-analysis of the association between neglected tropical diseases and malnutrition: more research needed on diseases other than intestinal parasites, leishmaniasis and leprosy. Access Microbiol..

[19] Crompton D.W., Nesheim M.C. (2002). Nutritional impact of intestinal helminthiasis during the human life cycle. Annu. Rev. Nutr..

[20] Rajagopal S., Hotez P.J., Bundy D.A. (2014). Micronutrient supplementation and deworming in children with geohelminth infections. PLOS Negl. Trop. Dis..

[21] Payne L.G., Koski K.G., Ortega-Barria E., Scott M.E. (2007). Benefit of vitamin A supplementation on Ascaris reinfection is less evident in stunted children. J Nutr.

[22] Tan P.Y., Loganathan R., Teng K.T., Lee S.C., Mohd Johari S.N., Selvaduray K.R. (2023). Red palm olein-enriched biscuit supplementation lowers Ascaris lumbricoides reinfection at 6-month after anthelmintic treatment among schoolchildren with vitamin A deficiency (VAD). Acta. Trop..

[23] Fauziah N., Aviani J.K., Agrianfanny Y.N., Fatimah S.N. (2022). Intestinal parasitic infection and nutritional status in children under five years old: a systematic review. Trop. Med. Infect. Dis..

[24] Davis E.L., Danon L., Prada J.M., Gunawardena S.A., Truscott J.E., Vlaminck J. (2018). Seasonally timed treatment programs for Ascaris lumbricoides to increase impact-an investigation using mathematical models. PLOS Negl. Trop. Dis..

[25] Harroff L.A., Liotta J.L., Bowman D.D., Angenent L.T. (2019). Current time-temperature relationships for thermal inactivation of Ascaris eggs at mesophilic temperatures are too conservative and may hamper development of simple, but effective sanitation. Water Res. X..

[26] Bekele F., Shumbej T. (2019). Fruit and vegetable contamination with medically important helminths and protozoans in Tarcha town, Dawuro zone, South West Ethiopia. Res. Rep. Trop. Med..

[27] Zeynudin A., Degefa T., Belay T., Mumicha J.B., Husen A., Yasin J. (2024). Parasitic contamination of fresh vegetables and fruits sold in open-air markets in peri-urban areas of Jimma City, Oromia, Ethiopia: a community-based cross-sectional study. PLoS One.

[28] Hall A., Hewitt G., Tuffrey V., de Silva N. (2008). A review and meta-analysis of the impact of intestinal worms on child growth and nutrition, Matern. Child Nutr..

[29] Mosites E., Dawson-Hahn E., Walson J., Rowhani-Rahbar A., Neuhouser M.L. (2017). Piecing together the stunting puzzle: a framework for attributable factors of child stunting. Paediatr. Int. Child Health..

[30] Mbuya M.N., Humphrey J.H. (2016). Preventing environmental enteric dysfunction through improved water, sanitation and hygiene: an opportunity for stunting reduction in developing countries. Matern. Child Nutr..

[31] Chao D.L., Roose A., Roh M., Kotloff K.L., Proctor J.L. (2019). The seasonality of diarrheal pathogens: a retrospective study of seven sites over three years. PLOS Negl. Trop. Dis..

[32] Ante-Testard P.A., Rerolle F., Nguyen A.T., Ashraf S., Parvez S.M., Naser A.M. (2024). WASH interventions and child diarrhea at the interface of climate and socioeconomic position in Bangladesh. Nat. Commun..

[33] Akinyemi P.A., Afolabi O.T., Aluko O.O. (2022). The effects of seasonal variations on household water security and burden of diarrheal diseases among under 5 children in an urban community, Southwest Nigeria. BMC Public Health.

[34] Baye K., Kennedy G. (2020). Estimates of dietary quality in infants and young children (6–23 mo): evidence from demographic and health surveys of 49 low- and middle-income countries. Nutrition.

[35] Krause R.J., Scott M.E., Sinisterra O.T., Koski K.G. (2023). Preschool child growth attainment and velocity during an agriculture intervention in rural Panama may be diminished by soil-transmitted helminths. Front. Public Health.

[36] Azriani D., Masita Q.N.S., Qinthara N.S., Yulita I.N., Agustian D., Zuhairini Y. (2024). Risk factors associated with stunting incidence in under five children in Southeast Asia: a scoping review. J Health Popul. Nutr..

[37] Islam B., Ibrahim T.I., Wang T., Wu M., Qin J. (2025). Current trends in household food insecurity, dietary diversity, and stunting among children under five in Asia: a systematic review. J Glob. Health..

[38] Crompton D.W. (1986). Nutritional aspects of infection. Trans. R Soc. Trop. Med. Hyg..

[39] Bhutta Z.A., Ahmed T., Black R.E., Cousens S., Dewey K., Giugliani E. (2008). What works? Interventions for maternal and child undernutrition and survival. Lancet.

[40] Tam E., Keats E.C., Rind F., Das J.K., Bhutta A.Z. (2020). Micronutrient supplementation and fortification interventions on health and development outcomes among children under-five in low- and middle-income countries: a systematic review and meta-analysis. Nutrients.

[41] Dewey K.G., Stewart C.P., Wessells K.R., Prado E.L., Arnold C.D. (2021). Small-quantity lipid-based nutrient supplements for the prevention of child malnutrition and promotion of healthy development: overview of individual participant data meta-analysis and programmatic implications. Am. J Clin. Nutr..

[42] Dessie G., Li J., Nghiem S., Doan T. (2025). Prevalence and determinants of stunting-anemia and wasting-anemia comorbidities and micronutrient deficiencies in children under 5 in the least-developed countries: a systematic review and meta-analysis. Nutr. Rev..

[43] Correia L.L., Rocha H.A., Campos J.S., Silva A.C., Silveira D.M., Machado M.M. (2019). Interaction between vitamin A supplementation and chronic malnutrition on child development. Cien. Saúde Colet.

[44] Sommer A. (1996). Uses and misuses of vitamin A. Curr. Issues Public Health.

[45] Djunaedi E., Sommer A., Pandji A., Kusdiono T.H.R., Taylor H.R. (1988). Impact of vitamin A supplementation on xerophthalmia: a randomized controlled community trial. Arch. Ophthalmol..

[46] Tarwotjo I., Sommer A., West K.P., Djunaedi E., Mele L., Hawkins B. (1987). Influence of participation on mortality in a randomized trial of vitamin A prophylaxis. Am. J Clin. Nutr..

[47] Stansfield S.K., Pierre-Louis M., Lerebours G., Augustin A. (1993). Vitamin A supplementation and increased prevalence of childhood diarrhoea and acute respiratory infections. Lancet.

[48] Robles-Sardin A.E., Astiazarán-García H., Dávalos-Navarro R., Quihui-Cota L., Cabrera-Pacheco R.M., Valencia M.E. (1998). Efecto de la suplementación con una dosis masiva de vitamina A en niños de 6 a 36 meses de edad [Effect of supplementation with a massive dose of vitamin A in children 6 to 36 months of age]. Salud Publica Mex.

[49] Pant C.R., Pokharel G.P., Curtale F., Pokhrel R.P., Grosse R.N., Lepkowski J. (1996). Impact of nutrition education and mega-dose vitamin A supplementation on the health of children in Nepal. Bull. World Health Organ..

[50] Ramakrishnan U., Latham M.C., Abel R., Frongillo E.A. (1995). Vitamin A supplementation and morbidity among preschool children in south India. Am. J Clin. Nutr..

[51] Underwood B.A. (1994). Hypovitaminosis A: international programmatic issues. J Nutr.

[52] Gordon B.L. (1982).

[53] Halpenny C.M., Koski K.G., Valdés V.E., Scott M.E. (2012). Prediction of child health by household density and asset-based indices in impoverished indigenous villages in rural Panamá. Am. J Trop. Med. Hyg..

[54] Pacific Disaster Center (2021). https://www.pdc.org/wp-content/uploads/Ngabe-Bugle.pdf.

[55] Ministerio de Salud Panamá (2025).

[56] Gibson R.S. (2005).

[57] Torheim L.E., Ouattara F., Diarra M.M., Thiam F.D., Barikmo I., Hatløy A. (2004). Nutrient adequacy and dietary diversity in rural Mali: association and determinants. Eur. J. Clin. Nutr..

[58] UNU/WHO/FAO (2004).

[59] UNU/WHO/FAO (2007). Protein and amino acid requirements in human nutrition: report of a joint FAO/WHO/UNU expert consultation, United Nations University, World Health Organization. Food and Agriculture Organization of the United Nations.

[60] FAO/WHO (1994).

[61] Patro-Golab B., Zalewski B.M., Kammermeier M., Schwingshackl L., Koletzko B. (2024). IUNS Task Force on Dietary Fat Quality, Current guidelines of fat intake in pregnancy and lactating women, infants, children and adolescents: a scoping review. Ann. Nutr. Metab..

[62] FAO/WHO (1998).

[63] Galicia L., de Romaña D.L., Harding K.B., De-Regil L.M., Grajeda R. (2016). Tackling malnutrition in Latin America and the Caribbean: challenges and opportunities. Rev. Panam. Salud Publica..

[64] Bekele T., Lachisa L., Tsegaye A., Bacha K., Ketema T. (2024). Efficacy of albendazole and mebendazole against soil transmitted infections among pre-school and school age children: a systematic review and meta-analysis. J Epidemiol. Glob. Health.

[65] Jia T.W., Melville S., Utzinger J., King C.H., Zhou X.N. (2012). Soil-transmitted helminth reinfection after drug treatment: a systematic review and meta-analysis. PLOS Negl. Trop. Dis..

[66] Gutiérrez-Jiménez J., Luna-Cázares L.M., Cruz L.M., de Aquino-López J.A., Sandoval-Gómez D., León-Ortiz A.T. (2019). Children from a rural region in the Chiapas highlands, Mexico, show an increased risk of stunting and intestinal parasitoses when compared with urban children. Bol. Med. Hosp. Infant. Mex..

[67] Yoseph A., Beyene H. (2020). The high prevalence of intestinal parasitic infections is associated with stunting among children aged 6–59 months in Boricha Woreda, Southern Ethiopia: a cross-sectional study. BMC Public Health.

[68] Brown K.H., Gilman R.H., Khatun M., Ahmed G. (1980). Absorption of macronutrients from a rice-vegetable diet before and after treatment of ascariasis in children. Am. J Clin. Nutr..

[69] Checkley W., Buckley G., Gilman R.H., Assis A.M., Guerrant R.L., Morris S.S. (2008). Multi-country analysis of the effects of diarrhoea on childhood stunting. Int. J Epidemiol.

[70] Richard S.A., Black R.E., Gilman R.H., Guerrant R.L., Kang G., Lanata C.F. (2013). Diarrhea in early childhood: short-term association with weight and long-term association with length. Am. J. Epidemiol..

[71] Mitra A.K., Wahed M.A., Chowdhury A.K., Stephensen C.B. (2002). Urinary retinol excretion in children with acute watery diarrhoea. J Health Popul. Nutr..

[72] Haider B.A., Bhutta Z.A. (2009). The effect of therapeutic zinc supplementation among young children with selected infections: a review of the evidence, Food Nutr. Bull..

[73] Penny M.E. (2013). Zinc supplementation in public health. Ann. Nutr. Metab..

[74] Ali A.A., Naqvi S.K., Hasnain Z., Zubairi M.B., Sharif A., Salam R.A. (2024). Zinc supplementation for acute and persistent watery diarrhoea in children: a systematic review and meta-analysis. J. Glob. Health..

[75] Shapiro M.J., Downs S.M., Swartz H.J., Parker M., Quelhas D., Kreis K. (2019). A systematic review investigating the relation between animal-source food consumption and stunting in children aged 6–60 months in low and middle-income countries. Adv. Nutr..

[76] Haile B., Headey D. (2023). Growth in milk consumption and reductions in child stunting: historical evidence from cross-country panel data. Food Policy.

[77] Hoppe C., Mølgaard C., Michaelsen K.F. (2006). Cow’s milk and linear growth in industrialized and developing countries. Annu. Rev. Nutr..

[78] Eaton J.C., Rothpletz-Puglia P., Dreker M.R., Iannotti L., Lutter C., Kaganda J. (2019). Effectiveness of provision of animal-source foods for supporting optimal growth and development in children 6 to 59 months of age. Cochrane Database Syst. Rev..

[79] Uauy R., Mize C.E., Castillo-Duran C. (2000). Fat intake during childhood: metabolic responses and effects on growth. Am. J. Clin. Nutr..

[80] Wyatt C.J., Triana Tejas M.A. (2000). Nutrient intake and growth of preschool children from different socioeconomic regions in the city of Oaxaca, México. Ann. Nutr. Metab..

[81] Barquera S., Rivera J.A., Safdie M., Flores M., Campos-Nonato I., Campirano F. (2003). Energy and nutrient intake in preschool and school age Mexican children: national Nutrition survey 1999. Salud Publica Mex.

[82] Monnard C., Fleith M. (2021). Total fat and fatty acid intake among 1–7-year-old children from 33 countries: comparison with international recommendations. Nutrients.

[83] Solomons N.W. (2024). Perspective on emerging micronutrient deficiencies in Latin America and the Caribbean. Food Nutr. Bull..

[84] Brito A., Cori H., Olivares M., Mujica M.F., Cediel G., López de Romaña D. (2013). Less than adequate vitamin D status and intake in Latin America and the Caribbean: a problem of unknown magnitude. Food Nutr. Bull..

[85] Cardino V.N., Goeden T., Yakah W., Ezeamama A.E., Fenton J.I. (2023). New perspectives on the associations between blood fatty acids, growth parameters, and cognitive development in global child populations. Nutrients.

[86] WHO (2015).

[87] Dewey K.G., Romero-Abal M.E., Quan de Serrano J., Bulux J., Peerson J.M., Engle P. (1997). A randomized intervention study of the effects of discontinuing coffee intake on growth and morbidity of iron-deficient Guatemalan toddlers. J Nutr.

[88] López de Romaña D., Olivares M., Brito A. (2015). Introduction: prevalence of micronutrient deficiencies in Latin America and the Caribbean. Food Nutr. Bull..

[89] Fernández-Gaxiola A.C., Neufeld L.M., García-Guerra A. (2024). Considerations for correction of micronutrient deficiencies through supplementation in pregnant women and children under-5 in Latin America. Food Nutr. Bull..

[90] van Stuijvenberg M.E., Nel J., Schoeman S.E., Lombard C.J., du Plessis L.M., Dhansay M.A. (2015). Low intake of calcium and vitamin D, but not zinc, iron or vitamin A, is associated with stunting in 2- to 5-year-old children. Nutrition.

[91] Khor G.L., Lee S.S. (2021). Complementary foods and milk-based formulas provide excess protein but suboptimal key micronutrients and essential fatty acids in the intakes of infants and toddlers from urban settings in Malaysia. Nutrients.

[92] Scatliff C.E., Koski K.G., Scott M.E. (2011). Diarrhea and novel dietary factors emerge as predictors of serum vitamin B12 in Panamanian children. Food Nutr. Bull..

[93] Pettifor J.M. (2014). Calcium and vitamin D metabolism in children in developing countries. Ann. Nutr. Metab..

[94] Hidayat K., Zhang L.L., Rizzoli R., Guo Y.X., Zhou Y., Shi Y.J. (2023). The effects of dairy product supplementation on bone health indices in children aged 3 to 18 years: a meta-analysis of randomized controlled trials. Adv. Nutr..

[95] Sheftel J., van Stuijvenberg M.E., Dhansay M.A., Suri D.J., Grahn M., Keuler N.S. (2022). Chronic and acute hypervitaminosis A are associated with suboptimal anthropometric measurements in a cohort of South African preschool children. Am. J Clin. Nutr..

[96] Nel J., van Stuijvenberg M.E., Schoeman S.E., Dhansay M.A., Lombard C.J., du Plessis L.M. (2014). Liver intake in 24-59-month-old children from an impoverished South African community provides enough vitamin A to meet requirements. Publ. Health Nutr..

