# Supplementary material for: Unbalanced Macronutrient Intakes, Multiple Micronutrient Inadequacies, and Diarrhea Underscore Low-Height-for-Age in Indigenous Panamanian Preschool Children
Source: Curr Dev Nutr. 2025 Sep 4;9(10):107547. doi: 10.1016/j.cdnut.2025.107547 (PMC12512998; doi:10.1016/j.cdnut.2025.107547)
Supplement: Multimedia component 1 [file mmc1.docx]

Supplementary Table S1. List of foods in diets of Ngäbe-Buglé preschool children.

| Leche KLIM | Tortilla de maíz | Chifa de Pifa | Guineo |
| --- | --- | --- | --- |
| Leche Nido | Bollo | Espinaca | Lentejas |
| Leche Évaporada | Tamales | Otoe | Porotos |
| Crema de maíz | Pescado | Ñame | Sanchocho de … |
| Chicheme | Pollo | Ñampi | Cacao |
| Pinolio | Carne | Calalu | Aceite |
| Huevos | Yuca | Piña | Dachin |
| Arroz | Plátano | Papaya |  |
| Pan | Pifa | Naranja |  |
